# Direct Observation of Early Stages of Growth of Multilayered DNA-Templated Au-Pd-Au Core-Shell Nanoparticles in Liquid Phase

**DOI:** 10.3389/fbioe.2019.00019

**Published:** 2019-02-26

**Authors:** Nabraj Bhattarai, Tanya Prozorov

**Affiliations:** Emergent Atomic and Magnetic Structures, Division of Materials Sciences and Engineering, Ames Laboratory, US Department of Energy, Ames, IA, United States

**Keywords:** bimetallic Au-Pd-Au core-shell nanostructures, nanoparticle growth, DNA-templated shell formation, liquid phase STEM *in situ*, dynamic analysis, low-dose imaging

## Abstract

We report here on direct observation of early stages of formation of multilayered bimetallic Au-Pd core-shell nanocubes and Au-Pd-Au core-shell nanostars in liquid phase using low-dose *in situ* scanning transmission electron microscopy (S/TEM) with the continuous flow fluid cell. The reduction of Pd and formation of Au-Pd core-shell is achieved through the flow of the reducing agent. Initial rapid growth of Pd on Au along <111> direction is followed by a slower rearrangement of Pd shell. We propose the mechanism for the DNA-directed shape transformation of Au-Pd core-shell nanocubes to adopt a nanostar-like morphology in the presence of T30 DNA and discuss the observed nanoparticle motion in the confined volume of the fluid cell. The growth of Au shell over Au-Pd nanocube is initiated at the vertices of the nanocubes, leading to the preferential growth of the {111} facets and resulting in formation of nanostar-like particles. While the core-shell nanostructures formed in a fluid cell *in situ* under the low-dose imaging conditions closely resemble those obtained in solution syntheses, the reaction kinetics in the fluid cell is affected by the radiolysis of liquid reagents induced by the electron beam, altering the rate-determining reaction steps. We discuss details of the growth processes and propose the reaction mechanism in liquid phase *in situ*.

## Introduction

Applications of nanoparticles (NPs) are determined by their shape, size, and crystalline structures (Sun and Xia, 2002; Tao et al., 2008; Xia et al., 2009; Ghosh Chaudhuri and Paria, 2011). Bimetallic core-shell (BM CS) NPs represent an important class of materials, where the synergetic combination of two different metals provides increased catalytic activity, selectivity, and stability. Decorating the nanoparticle core with the catalytically active precious metals allows reducing the cost of catalysts. By following some specific synthesis procedures control over the shape, size, and crystalline structures can be achieved (Alayoglu et al., 2008; Tao et al., 2008; Ghosh Chaudhuri and Paria, 2011). While it is generally accepted that the exploitable properties of BM CS NPs can be controlled during the seed-mediated synthesis, despite extensive research effort, the exact mechanism of shell formation and growth over the core remains unclear (Ferrer et al., 2007; Alayoglu et al., 2008; Lee et al., 2009; Yang et al., 2011; Ross, 2015). In particular, existing reports on seed-mediated core-shell synthesis do not address the early stages of the formation and growth of the shell, critical in determining the morphology of resultant nanostructures. The *in situ* fluid cell electron microscopy (EM) is a powerful technique uniquely suitable for the visualization of the formation and growth of NPs in real time and correlating the changes in NP size and shape with the evolution of its crystalline structure (Liao et al., 2012; Kashyap et al., 2014; Alloyeau et al., 2015; Chen Q. et al., 2015; Chen X. et al., 2015; Gamalski et al., 2015; Hellebusch et al., 2015; Hermannsdörfer et al., 2015; Ievlev et al., 2015; Liang et al., 2015a,b; Nagao et al., 2015; Niu et al., 2015; Ross, 2015; Weiner et al., 2016). Recent reports on *in situ* scanning/transmission electron microscopy (S/TEM) characterization employing gas and liquid cell holder platforms highlighted advances in high-resolution imaging and precise quantification of the particle growth and mobility and pointed to the importance of controlling the electron dose (Williamson et al., 2003; Yuk et al., 2012, 2016; Welch et al., 2013, 2015; Abellan et al., 2014; Lewis et al., 2014; Liao et al., 2014; Woehl et al., 2014; Liu et al., 2015; Ngo and Yang, 2015; Park et al., 2015b,c; Patterson et al., 2015; Pohlmann et al., 2015; Stehle et al., 2015; Woehl and Prozorov, 2015).

The majority of published studies on the nucleation and growth of NPs in liquid phase *in situ* with the S/TEM utilized electron beam as a sole reducing agent, with the reducing reaction conditions generated *via* the interaction of electron beam with the precursor and solvent (Jungjohann et al., 2013; Sutter and Sutter, 2014). In a majority of reported cases, the kinetics of the reactions induced by the interaction of hydrated electrons with the surrounding liquid, differs from the actual reaction kinetics taking place in the bulk synthesis. It is worth noting that the shape, size, and crystalline structures can also be affected by the electron beam dose and the chemistry of the radiolyzed liquid reagents (Abellan et al., 2014). As a result, the comparison between the *in situ* and *ex situ* experiments is not straightforward. Recently, the growth mechanisms of Au-Pd BM CS NPs were studied in liquid phase with the static cell, using hydrated electrons (Jungjohann et al., 2013; Sutter and Sutter, 2014). In addition to the closed/static cell experiments, Wu et al. reported the growth of Au on icosahedral Pt seed using a liquid cell holder, and showed that kinetically controlled growth led to diffusion of Au from corner islands to terraces and edges during the growth process (Wu et al., 2015). Importantly, since Au precursor and citric acid used in these studies were flown into the reaction chamber, in all likelihood it resulted in the reduction of Au salt occurring *before* the imaging commenced. The *in situ* imaging would, most likely, miss the initial steps of formation of nascent shell, and the system, therefore, would not be entirely representative of the early reaction conditions of the solution synthesis.

While there is a wealth of information on the NPs formation available in the literature, the movement of particles with respect to SiN window, in terms of both translational and rotational modes, remains not well-understood. Recently, Weiner et al. reported the NPs to be attached to SiN *via* frictional forces between the particles and window membranes (Weiner et al., 2016). Such an attachment would inhibit translational motion of NPs and render them largely immobile. Similarly, Park et al. reported the tracking of single rotating particle in graphene liquid cell using 3D tomography (Park et al., 2015a). However, there is still lacking a clear understanding of a rotational and wobbling movement phenomena of individual nanoparticle deposited onto a SiN window membrane.

Here, we report on direct observation of the early stages of growth of Pd shell on Au nanoparticle core using the high angle annular dark field (HAADF) mode under the low dose STEM imaging conditions in liquid phase. In order to visualize the growth processes occurring *in situ*, we focused on creating a near-native reaction environment such a way as to minimize the reductive action of the electron beam. In our work, this was achieved by a controlled flow of the Pd precursor, following by the delivery of a reducing agent (ascorbic acid, AA) into the liquid cell. The continuous flow of liquid through the reaction chamber allowed maintaining the fresh environment inside the reaction chamber, while minimizing the effects of radiolysis (Ngo and Yang, 2015; Wu et al., 2015). A series of *in situ* low-dose imaging experiments, including static and continuous flow setup, is presented in Supplementary Material. The wobbling motion of individual particle is also discussed.

## Materials and Methods

Potassium tetrachloropalladate (K_2_PdCl_4_, 98%), gold (III) chloride trihydrate (HAuCl_4_ × 3H_2_O, 99.9% trace metal basis), hexadecyltrimethylammonium bromide (CTAB, 99%), and L-ascorbic acid (AA, 99%) were purchased from Sigma Aldrich and used as received. All aqueous solutions were prepared with deionized water passed through a Millipore Milli-Q Plus water purification system (ρ = 18.2 MΩ cm).

### Solution Synthesis

#### Au Nanoparticle Synthesis

Au nanoparticles (NPs) were synthesized using seed mediated growth process following the procedure reported in the literature^1−3^. Larger Au NPs with the size of ~50 nm, were synthesized by mixing 5 mL of Au NPs (~15 nm sized) with HAuCl_4_ (25 mM, 0.2 mL) and reducing them using ascorbic acid (AA) (0.1 M, 0.5 mL).

#### Synthesis of Au-Pd Core-Shell NPs

Au-Pd core-shell NPs were obtained by adding Pd precursor [K_2_PdCl_4_ (10 mM, 1 mL)] onto larger Au NPs and reducing by AA (0.1 M, 0.5 mL). The size of the shell formed on the nanoparticle was controlled by the amount of Pd introduced into the reaction. Conventional S/TEM samples were prepared by placing a 3 μL droplet of aqueous colloidal nanoparticles suspension onto a Quantifoil carbon grid for each step and dried at room temperature.

#### S/TEM Characterization

The STEM images were recorded for each step using FEI Tecnai G^2^ F20 (S)TEM equipped with a Tridium Gatan image filter operating at 200 kV, used in the high annular dark field (HADF) STEM mode. Continuous capture movies were recorded for ~3 min using a freeware screen grabber, AutoScreenRecorder (Wisdom Software) that recorded images at a rate of 8 frames per second. Determining dose-rate and critical dose on the specimen was conducted at the Center for Nanophase Materials Sciences, Oak Ridge National Laboratory with the FEI Titan-S aberration-corrected TEM/STEM equipped with a Gatan Quantum EELS and Gatan Imaging Filter (GIF), with dual-EELS and fast spectrum imaging capabilities, Gatan *OneView* CMOS camera with *in-situ* option for high-frame-rate image/video capture. Continuous capture movies were recorder using a Camtasia Studio 8.0 screen recorder and video editor software (TechSmith). The Titan-S aberration-corrected TEM/STEM operated at 300 kV and was used in the HAADF-STEM mode. The work on this instrument was performed with using a specialized liquid-flow holder (Protochips *Poseidon*). Data analysis was performed with ES Vision (FEI) software (ES Vision version 5.0) and OriginPro 9.0 software. Video editing was performed with BigaSoft Total Video Convertor (Bigasoft 6).

### *In situ* Fluid Cell STEM Imaging

#### Fluid Cell Assembly

The *in situ* liquid cell STEM experiment was carried out using a commercial continuous flow fluid cell holder platform (Hummingbird Scientific, Lacey, WA, USA). Silicon nitride chips were UV/O_3_ plasma-cleaned using Pro-cleaner (Bioforce Nanosciences, Ames, IA, USA) for 30 min prior to use to hydrophilize the surfaces and ensure contaminant removal. A thin liquid layer (typically 100–300 nm thick) was formed by sandwiching two SiN coated silicon chips with a 50 × 200 μm electron transparent 50 nm thick SiN opening etched from the center, forming imaging window. In the continuous flow cell experiments, both SiN windows had a 100 nm SU- 8 spacer. In the static cell experiments, only one SiN window had a 100 nm SU-8 spacer, while the other had no spacer used. The liquid layer and silicon chips were sealed to prevent evaporation of the liquid. In all experiments described in this work, nanoparticles were deposited on the chip that was assembled on top of the cell, while the bottom chip was loaded liquid- and particle-free. To allow the majority of nanoparticles to adhere to the top SiN window, we drop casted 0.5 μL of the nanoparticle solution on the top SiN window and allowed the solution to remain on the surface for ~3 min, covered it to avoid evaporation, gently bloated the excess of the liquid with lens paper, and pairing with the second chip to form the liquid layer. For Au-Pd DNA experiments, 0.5 μL of the NPs aqueous suspension (Au-Pd core-shell nanocubes incubated with 10 μL of T30 DNA) were placed onto the chip and assembled at the top of the liquid cell, with the excess of liquid being gently bloated away with the lens paper. Following the cell assembly, the hermetically sealed holder was inserted into the microscope and allowed to equilibrate for 20 min before imaging in the HAADF STEM mode. *In situ* fluid delivery was carried out with a syringe pump (Hummingbird Scientific, Lacey, WA, USA) operating with a variable pumping speed (2–5 μL/min).

#### *In situ* Liquid Phase Imaging With the Static Cell

Au octahedral nanoparticles (~12 nm) were mixed with K_2_PdCl_4_ (2 μL, 0.5 mM) and AA (2 μL, 5 mM). 0.4 μL of this mixture were deposited onto the top square chip with a window paired with a 100 nm spacer chip on the bottom. Excess liquid was gently bloated away with lens paper. The time allotted after mixing Au nanoparticles, Pd precursor and AA and imaging was ~50 min.

#### *In situ* Liquid Phase Imaging With the Continuous Fluid Flow Cell: Delivery of Ascorbic Acid to the Au Nanoparticle Seeds Incubated With the Pd Precursor

To aid in the visualization of growth of core-shell structures while maintaining the low-dose imaging conditions, the *in situ* growth experiments were carried out with ~40 nm Au seeds. Au nanoparticles were mixed with the Pd precursor (2 μL, 0.5 mM). 0.4 μL of this mixture was deposited onto the window with a 100 nm spacer chip, topped with another 100 nm spacer chip window and sealed. After assembly and vacuum check, AA (0.1 M, 100 μL) was flowed into the reaction chamber at 5 μL/min and the changes in the structures morphology were recorded by using HAADF STEM imaging *in situ*. Total time after mixing Au NPs and Pd precursors and imaging was ~90 min, which included the periods of time between mixing the reagents before cell assembly and holder stabilization, before the delivery of AA. The electron dose delivered to the specimen was calculated at 0.146 e^−^/(Å^2^·s).

#### *In situ* Liquid Phase Imaging With the Continuous Fluid Flow Cell: Sequential Delivery of Pd Precursor and Ascorbic acid Solutions to the Fluid Cell Chamber

At least 10 individual nanoparticles were examined, their positions recorded, and images acquired. Unless stated otherwise, all *in situ* imaging was carried out using the following conditions: CLA #70, spot size 11, I_e_ = 8 *p*A, dwell time = 8 μs acquisition, scanning time = 2 μs, total frame time t_f_ = 10.06 s, 56,000 X magnification. The electron dose rate was calculated at 0.146 e^−^/(Å^2^·s).

#### Delivery of Pd Precursor and Ascorbic Acid

After imaging initial positions of 10 arbitrary chosen Au nanoparticles, K_2_PdCl_4_ precursor (5 mM, 100 μL) was flown into the reaction chamber at 2.5 μL/min, allowed to react for 30 min, and then previously recorded 10 positions were imaged at the same magnification settings. Nanopure water (2.5 μL/min, 35 μL) was flown through to displace trace amounts of Pd precursor from the fluid line and discarded without entering the liquid cell chamber. Following this cleaning step, ascorbic acid (AA, 0.1 M, 100 μL) was flown into the liquid cell chamber at 2.5 μL/min and STEM imaging was continued at previously recorded positions. The investigation was carried out after 30 min of introducing AA into the liquid cell chamber with periodic image acquisition during the flow of AA. The observed changes were video recorded and subsequently HAADF STEM images were also acquired.

## Results

**Figure 1:**
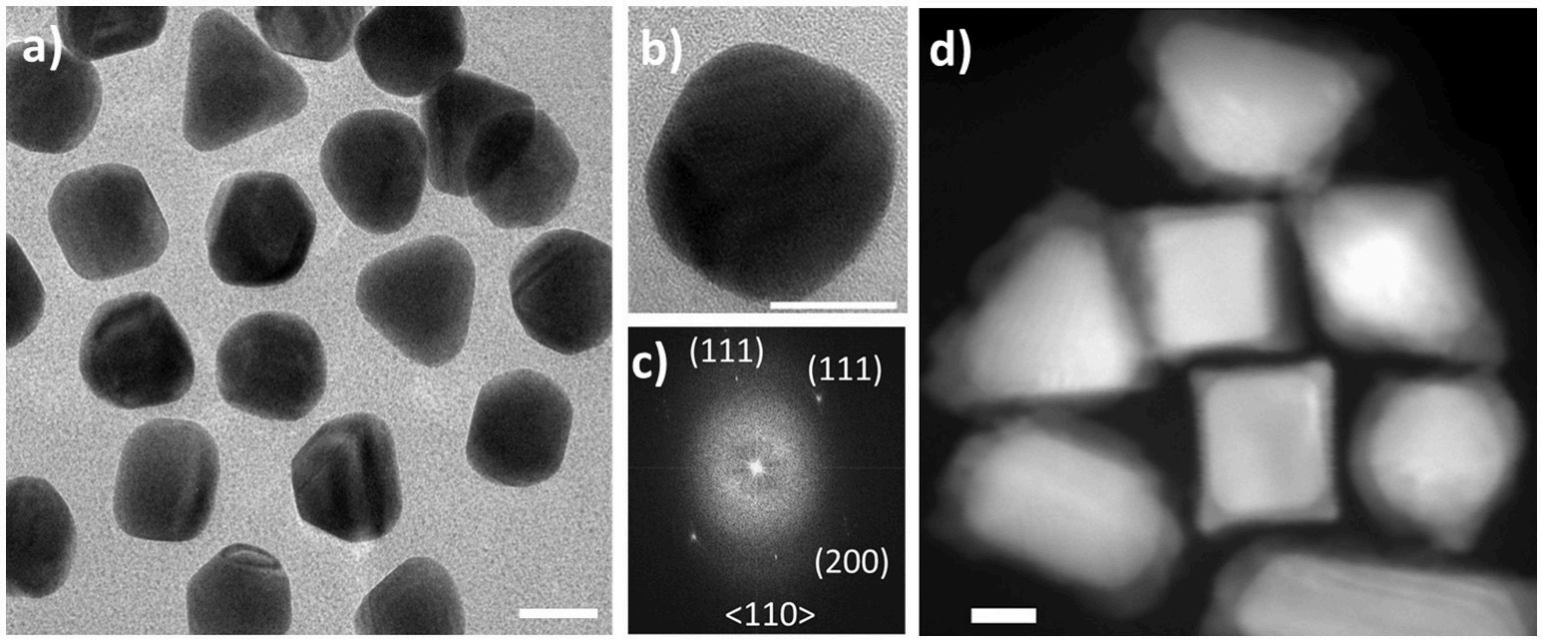
Au seeds and Au-Pd core-shell nanoparticles. **(A)** BF TEM micrographs of Au nanoparticles with the sizes 40 ± 5 nm with mostly octahedral morphology used as seeds in core-shell synthesis; **(B)** typical octahedral Au nanoparticle with **(C)** FFT pattern used in lieu of the electron diffraction pattern; **(D)** HAADF-STEM image of Au-Pd core-shell nanoparticles grown from **(A)** *via* the seed-mediated growth process in the presence of ascorbic acid. Scale bars: 20 nm.

The Au and Au-Pd CS NPs (Supplementary Figures 1A,B, Supplementary Material) were synthesized following the procedure described elsewhere (Bhattarai et al., 2013b; Bhattarai and Prozorov, 2015). Briefly, Au NPs (~15 nm) were grown into 40 ± 5 nm-sized NPs (Figure 1A) with addition of excess Au and reduced by AA. The Au NPs obtained are mostly octahedral, with occasional trigonal prism morphologies. A typical ~40 nm-sized octahedral Au NP is presented in Figure 1B, along with the corresponding fast Fourier transform (FFT) (Figure 1C) used in lieu of the electron diffraction pattern. The use of these relatively large particles allowed the imaging at lower magnification, so that low dose STEM conditions could be maintained during *in situ* investigation. The obtained Au-Pd NPs shown in Figure 1D, exhibited cubic, trigonal prismatic, and rod-like CS morphologies. The Au-Pd CS nanocubes formed *via* the bulk synthesis, with the thickness of Pd shell of 12 ± 1 nm are shown in Supplementary Figure 1B. Several research groups reported on *ex situ* studies aimed at understanding the growth mechanism of the formation of Au-Pd core shell nanocubes from Au octahedral NPs (Xiang et al., 2006; Yang et al., 2011; Bhattarai et al., 2013a). It is generally assumed that Br^−^ ions from CTAB are adsorbed onto surfaces of the octahedral NP seeds, resulting in a fast growth along <111> directions upon addition of Pd, and the formation of stable (100) surfaces takes place forming CS nanostructures (Lewis et al., 2014).

**Figure 2:**
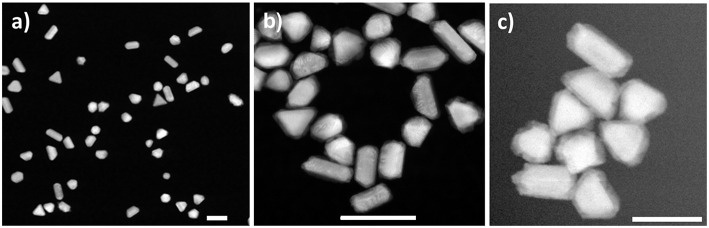
HAADF-STEM images of nanoparticles acquired in this study. **(A)** Au seeds used in bulk synthesis exhibiting various morphologies*;* **(B)** Au-Pd core-shell nanoparticles obtained in solution synthesis and imaged upon drying; **(C)** Au-Pd core-shell nanoparticles formed and imaged in liquid phase after delivering ascorbic acid while maintaining the dose rate estimated at 1.2 e^−^/(Å^2^·s). Scale bars: 100 nm.

**Figure 3:**
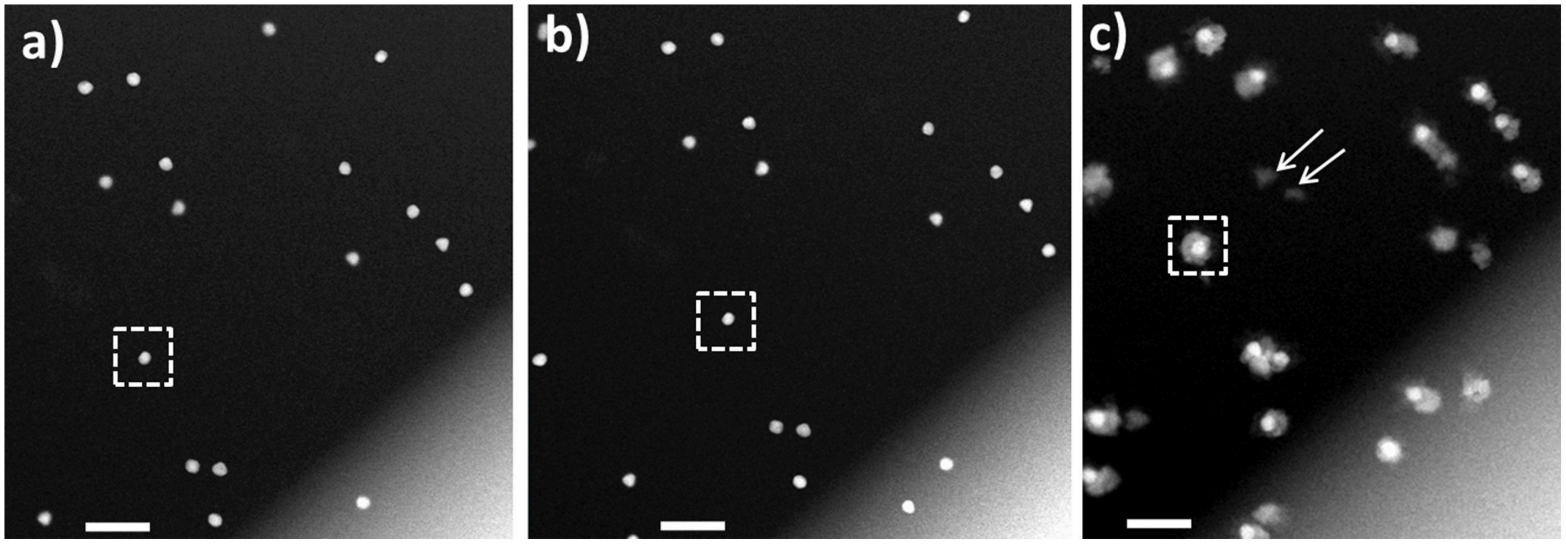
HAADF-STEM imaging in liquid phase. **(A)** Au nanoparticle seeds lodged between the two SiN window membranes; **(B)** same area after flowing Pd precursor; **(C)** Au-Pd core-shell nanostructures formed after flowing AA into the fluid cell. The Au NP denoted with the dotted square in **(A,B)** transforms into a symmetrical core-shell structure in **(C)**. Arrows mark self-nucleated Pd nanoparticles. Scale bars: 200 nm.

To investigate the CS growth mechanism, we carried out the *in situ* imaging experiments utilizing both static and continuous flow fluid cell configurations. In the *in situ* static imaging experiment, all reagents were mixed and sealed between the two SiN window membranes prior to the imaging. The liquid cell reaction visualized in the static mode (Supplementary Figure 2C) yielded the CS NPs with well-defined shapes and sizes. Based on the differences in the Z-contrast used in HAADF-STEM imaging mode, the core is composed of a higher atomic number element (Au), while the formed shell is comprised of a lower atomic number element (Pd). The shapes and sizes of the nanocubes (Supplementary Figure 2C) are in good agreement with the results obtained *ex situ* (Supplementary Figure 2B), which is indicative of achieving comparable reaction conditions in both cases. Notably, while the results confirmed the feasibility of generating the reaction environment *in situ* conducive of the seed-mediated core-shell formation in the presence of AA, the CS structures were evidently formed before the imaging has begun. As a result, the early steps of the shell formation and growth taking place during the reaction could not be visualized with the static cell. Therefore, to elucidate the seed-mediated growth mechanism of CS NPs formation from Au NPs and visualize the reaction in real time, further *in situ* imaging experiments were carried out using the continuous fluid flow cell with a controlled delivery of the reactants. In doing so, we avoided the undesired pre-imaging formation of Pd shell on the Au seeds. In the course of *in situ* experiments with the continuous flow fluid cell, the formation of the Pd shell over the initial Au seeds was monitored continuously, while delivering the reagent solutions into the reaction fluid cell and imaging each step of the reaction. *in situ* movie, detailed about the cell assembly and experimental conditions are presented in the experimental section in SI. Figure 2A shows the Au seed NPs used for the *in situ* Au-Pd CS growth experiments. We used the specimen with varied shapes consisting of nanorods, trigonal prisms, and cubes, to elucidate the shape-specific Pd shell formation along the different crystallographic directions. The CS structures obtained in bulk synthesis *ex situ* (Figure 2B) exhibited Pd shell formed around the Au core, with the most isotropic growth observed in trigonal prisms, and more anisotropic growth observed for other shapes. In our *in situ* CS formation experiments, aqueous Pd solution was mixed with Au seed NPs sealed in the fluid cell and allowed to mix, followed by the delivery of AA into the reaction chamber and Pd reduction takes place forming Au-Pd CS nanostructures *in situ* (Figure 2C), closely resembling those obtained in the *ex situ* experiments (Figure 2B). Despite close resemblance of the structure in *ex situ* and *in situ*, the early stages of the formation were missing, when the imaging was carried out, the particles were already formed. Here the acquired electron dose used was calculated to be 1.2 e^−^/(Å^2^). For additional details see the experimental conditions in Supplementary Material. Figure 3 shows the images obtained from in liquid phase under continuous flow reaction conditions. The HAADF-STEM imaging of Au NPs in the reaction chamber (Figure 3A), reveals uniform NPs with the sizes of ~40 ± 5 nm. Ten different positions were recorded on the stage at different areas of the reaction chamber near the edges of the window assuming that the liquid thickness to be the lowest near the edges. After the imaging, Pd precursor was flown into the reaction chamber, allowed to react, and imaged again at the previously recorded positions.

An example of the HAADF-STEM imaging after flowing Pd precursor is presented in Figure 3B, where no significant changes in the morphology were noted, and no Pd ions deposited onto Au NPs were detected. Afterwards, AA was flown into the chamber, followed by the STEM imaging at the selected, previously recorded positions. Use of this setup ensured avoiding the undesired reduction of precursor before reaching to the reaction chamber, which was not mentioned in recent work by Wu et al. (2015). The deposition of Pd shell on the Au core was observed and an example is presented in Figure 3C. For additional details we refer the Reader to Video 1 in Supplementary Material. The HAADF-STEM imaging permits observation of Pd deposition in shell on Au core forming core-shell structure. During the shell formation, some Au atoms (surface free energy of Au is 1.63 J/m^2^) from a core will diffuse to a Pd shell (surface free energy of Pd is 2.04 J/m^2^) in order to minimize the surface free energy, as reported in early *ex situ* studies (Mezey and Giber, 1982; Ding et al., 2010). It is pertinent to mention, that image resolution obtained from low dose STEM conditions imposed in our study, is not sufficient to see the diffusion of Au or Pd atoms. Further, the repeated imaging for same region and continuous exposure of electron beam for both imaging and video recording results higher cumulative electron dose. The cumulative electron dose for Figure 3C was estimated at 70 e^−^/Å^2^.

**Figure 4:**
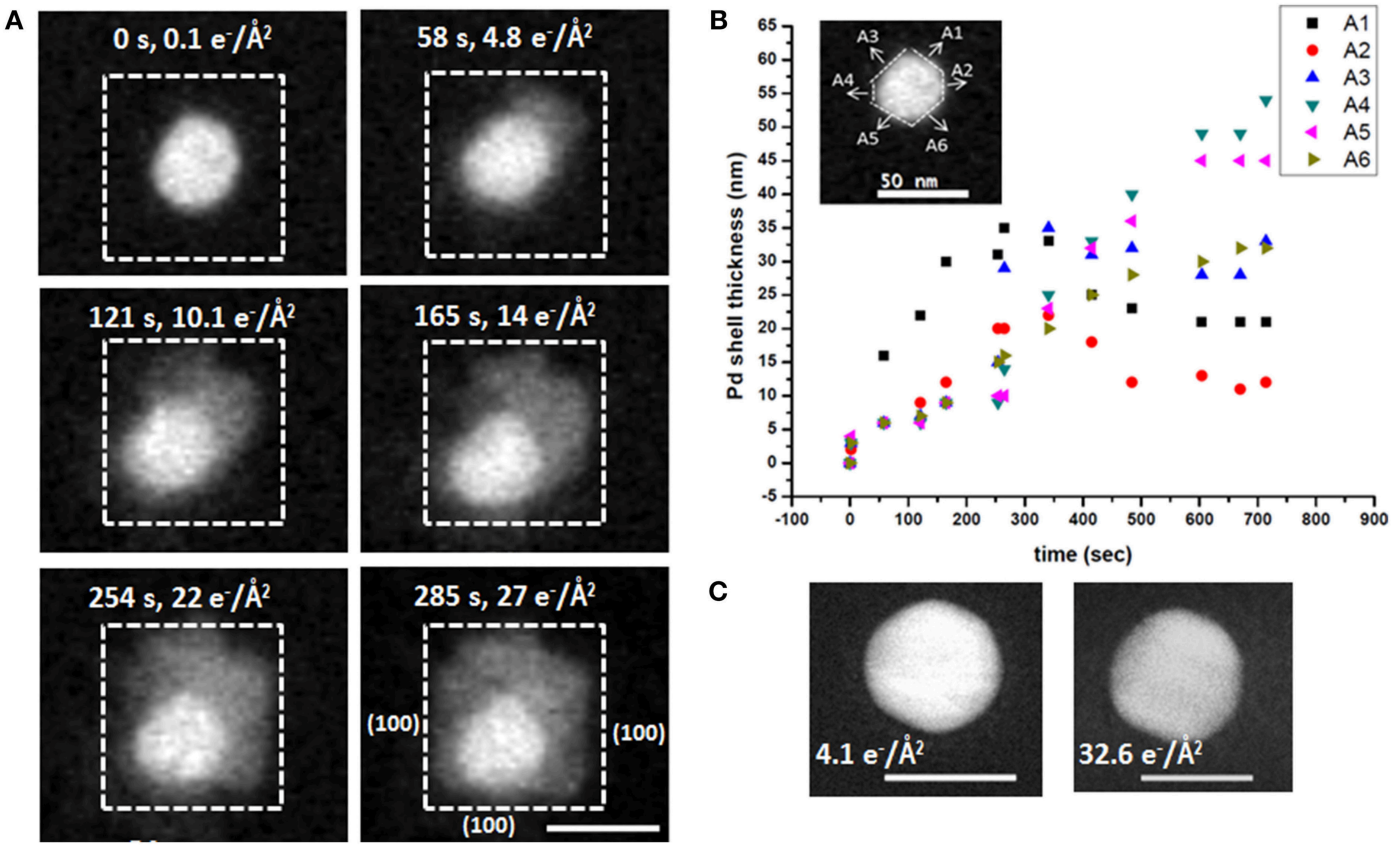
Analysis of the Au-Pd core-shell nanostructure formation process shown in Figure. **(A)** HAADF-STEM images of the growth of Pd shell on the individual Au nanoparticle after flowing ascorbic acid acquired at specific imaging times and shown together with the corresponding electron doses; **(B)** Thickness of the formed Pd shell as a function of time after AA delivery, measured along the six directions (A1–A6, inset); **(C)** The lack of growth of Pd shell in the absence of AA, with the accumulated electron dose estimated at 32.6 e^−^/Å^2^ (right), shown in comparison to the same particle at the accumulated electron dose of 4.1 e^−^/Å^2^ (left). Scale bars: 50 nm.

To follow the growth of individual particle, the Au NP represented by white dotted square in Figure 3A is considered a typical example, with selected snapshots of zoomed in STEM images of that NP acquired at the dose rate of 0.1 e^−^/(Å^2^·s) shown in Figure 4A. Here the thickness of the formed Pd shell is represented by the distances measured along the six arbitrary directions, A1 to A6, as shown by the inset in Figure 4B. During the first 58 s of imaging, Pd shell grows most rapidly along A1 direction reaching the thickness of 16 ± 1 nm, while the growth along other directions remains almost constant and yields ~6 ± 1 nm thickness. At 121 s, the thickness of Pd layer increases continuously along A1 direction and reaches 22 ± 1 nm, which is accompanied by the more pronounced growth along A2 at 9 ± 1 nm, while remaining unchanged at 6 ± 1 nm along all other directions. Similarly, at 165 s, the shell thickness grown along A1 direction is 30 ± 1 nm, while being 12 ± 1 nm along A2, and along A3, A4, A5, and that of 9 ± 1 nm along A6, respectively. Overall, the thickness of Pd shell increases with time. At 254 s, its growth along A1, A2, A3, A4, A5, and A6 measured to be 31 ± 1, 20 ± 1, 15 ± 1, 9 ± 1, 10 ± 1, and 15 ± 1 nm, respectively. Similarly, at 285 s, the growth along A1 yields the shell with the thickness of 35 ± 1 nm, A2 is 20 ± 1 nm, A3 is 29 ± 1 nm, A4 is 14 ± 1 nm, A5 is 10 ± 1 nm, and A6 is 16 ± 1 nm, respectively. The electron dose during imaging is calculated and plotted with the Pd shell thickness, as shown in Figures 4B,C. Here the growth of Pd shell over Au core occurs at low dose rate [0.1 e^−^/(Å^2^·s)] (Figure 4B), consistent of generating in the reaction chamber the conditions conducive to the reduction of Pd. Notably, no growth of Pd shell could be detected in the absence of AA at 32.6 e^−^/Å^2^ (Figure 4C), implying that the conditions conducive for the Pd reduction were not met in this case.

## Discussion

The rapid growth of Pd shell along the <111> direction is consistent with the preferential chemisorption of CTAB on the {100} crystal facets of Au NPs reported in the literature (Jana et al., 2001; Gao et al., 2003; Xiang et al., 2006; Sun et al., 2007; Niu et al., 2008). We believe this process to involve a rapid deposition of positively charged Pd ions and resultant formation of a disordered juvenile Pd shell along <111> direction, consistent with a subtle variation of the Z-contrast throughout the shell in the HAADF-STEM images in Figure 4A (0–165 s). The subsequent slower rearrangement of the newly formed Pd shell produces denser, more ordered structure, as manifested by the decrease of thickness along A1 and A3 noted at 254 s, as well as by a slight enhancement of the overall Z-contrast (254–285 s). Following this ordering, the Pd (100) surface is stabilized to form the nanocube and yields a core-shell Au-Pd structure. This direct visualization of growth is in good agreement with reports on *ex situ* studies (Bhattarai et al., 2013a). Every effort was made to remain under the low-dose imaging conditions, and so we resorted to working at lower magnification, and hence were not able to observe the Pd deposition with atomic resolution. In addition, the rotation or wobbling of each particle cannot be tracked at low magnification. The HAADF STEM images and also movie implies that there is no translational motion or movement of individual particles. This observation is in agreement with recent report where the attachment of NPs in SiN membrane inhibits the translational motion (Weiner et al., 2016). We further investigated the rotation or wobbling of each nanoparticle during the growth process. We digitally zoomed into 6 different NPs as represented by 1–6 in **Figure 7A** in each frame and measured the angle between arbitrary facet with the base line as shown in Supplementary Figure 3. The angle measured on each frame is subtracted from the initial angle and this difference in angle is plotted with corresponding time as shown in **Figure 7B**. This plot shows that all particles are wobbling by some angles, some wobbles by more others by less. For example, particle 1 quickly rotates initially for <100 s after that it wobbles by small angles. Similarly, other particles 2,3,4,5, and 6 wobble back and forth.

**Figure 5:**
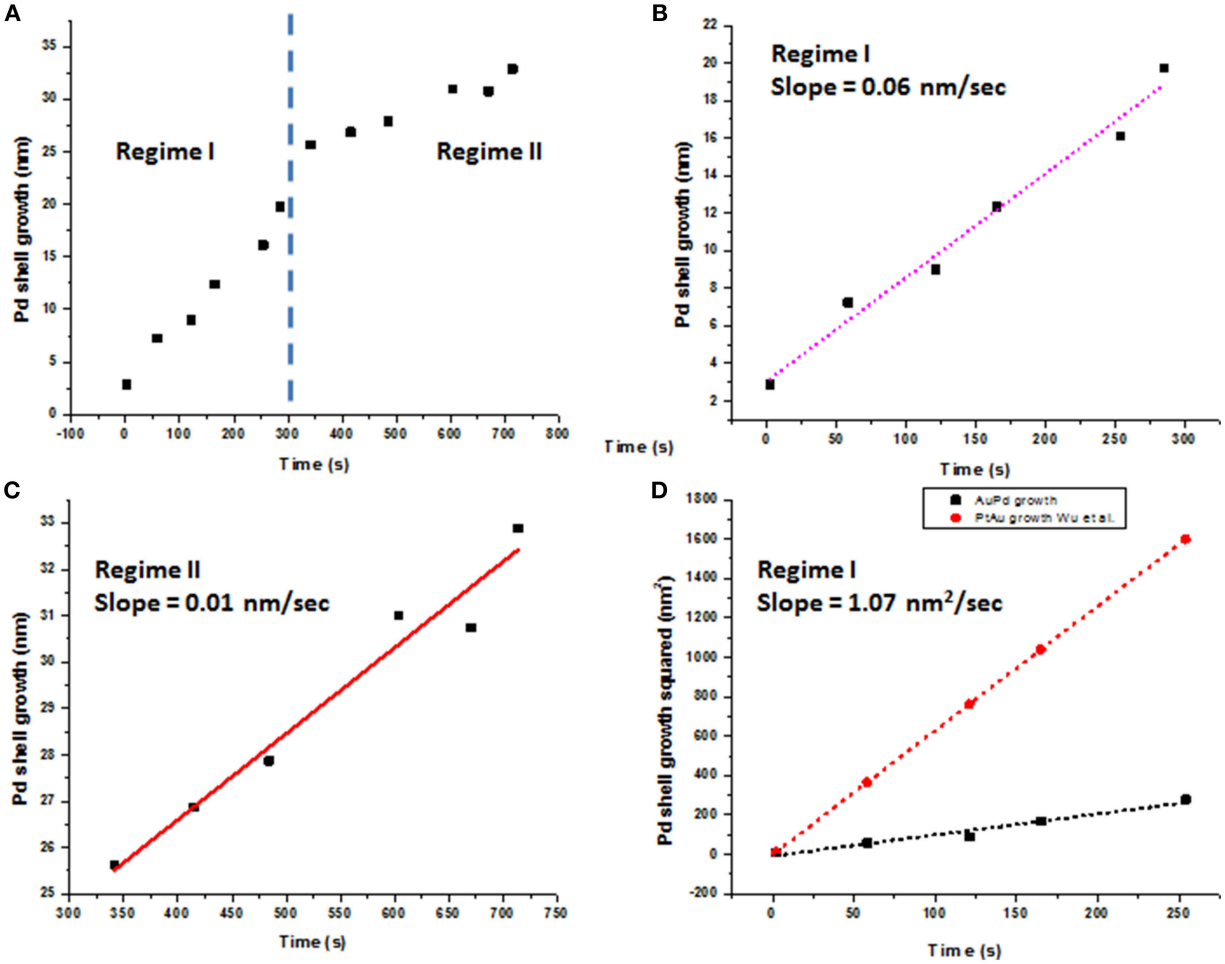
Pd shell growth plotted as a function of time. **(A)** Pd shell growth exhibits two distinctly different growth rates, denoted as **Regime I** and Regime II; **(B)** Linear plot of Pd shell thickness (nm) vs. time (s) in Regime I with a slope of 0.06 nm/s; **(C)** linear plot of Pd shell thickness (nm) vs. time (s) for **Regime II** with a slope of 0.01 nm/s; **(D)** Growth rate in **Regime I** fitted as d^2^ = K_R_t for the reaction-limited growth, with K_R_ value 1.07 nm^2^/s. Au-Pt growth from the recent report by Wu et al. (2015) is shown for comparison.

The average Pd shell growth plotted as a function of time is shown in Figure 5. The *in situ* data revealed two-step growth with distinctly different growth kinetics, as shown in Figure 5A. We hypothesize that the processes taking place in **Regime I** correspond to a rapid beam-enhanced reduction of Pd and steady deposition of Pd shell onto Au core, with the epitaxial growth made possible by a low (4.7%) lattice mismatch Au and Pd (Ding et al., 2010). **Regime II** covers complex phenomena associated with redistribution and rearrangement of a surplus of rapidly reduced Pd away from the Au-Pd interface to align with a crystallographic orientation of the already formed Pd surface. The growth kinetics in both regimes was analyzed using Lifshitz-Slyozov-Wagner (LSW) model following the recent reports (Dirksen and Ring, 1991; Murray et al., 2000; Wu et al., 2015). Here the diffusion-limited growth model (Jian et al., 2002; Rao et al., 2007) yields the calculated growth rate of ~55 nm/s, roughly 3 orders of magnitude higher than the experimentally observed values, implying that the diffusion-limited growth does not take place. Furthermore, assuming the reaction-limited growth rate fitted with the value of K_R_ estimated at 1.07 nm^2^/s (Figure 5D), the reaction rate do not follow any one specific reaction rate law, and best described by the hybrid growth reaction-diffusion limited growth in **Regime I**.

The **Regime II**, with the growth rate of ~0.01 nm/s (Figure 5C), is associated with a *remodeling* of the Pd shell, now concurrent with the continuous precipitous deposition of palladium produced both *via* the reaction with AA and that of direct reduction with the electron beam. We hypothesize, that in our experimental setup **Regime II** becomes a rate-determining step. The initial deposition of Pd and formation of the nascent Pd shell over Au core occur more readily along the <111> crystallographic direction than along <100> crystallographic direction. After the forming Pd layer covers the Au-Pd interface, sustaining the prolonged anisotropic, “pointed” rapid growth along the <111> direction becomes energetically unfavorable and yields to a slower remodeling of the face of Pd shell. While such a remodeling is likely to occur in all reactions, it does not become a rate-determining step in the bulk synthesis. We further hypothesize, that continuous rapid deposition of reduced Pd from solution leads to accumulation of disordered Pd on the formed Pd surface, which, in turn, undergoes ordering in a process similar to oriented attachment growth (Liao et al., 2012). At this stage, the added Pd is a subject to continuous rotation movement over the present Pd layer, until the energetically favorable lattice sites are found and the surface free energy is reduced. As the straightening and mass redistribution of Pd take place, the thickness of Pd shell decreases from the corners and equilibrates to increase toward other directions, as seen in Figure 4B. The diffusion of Pd over Pd (100) surfaces could also be facilitated by the bridge hopping mechanism described by Perkins and co-authors (Perkins and DePristo, 1993).

**Figure 6:**
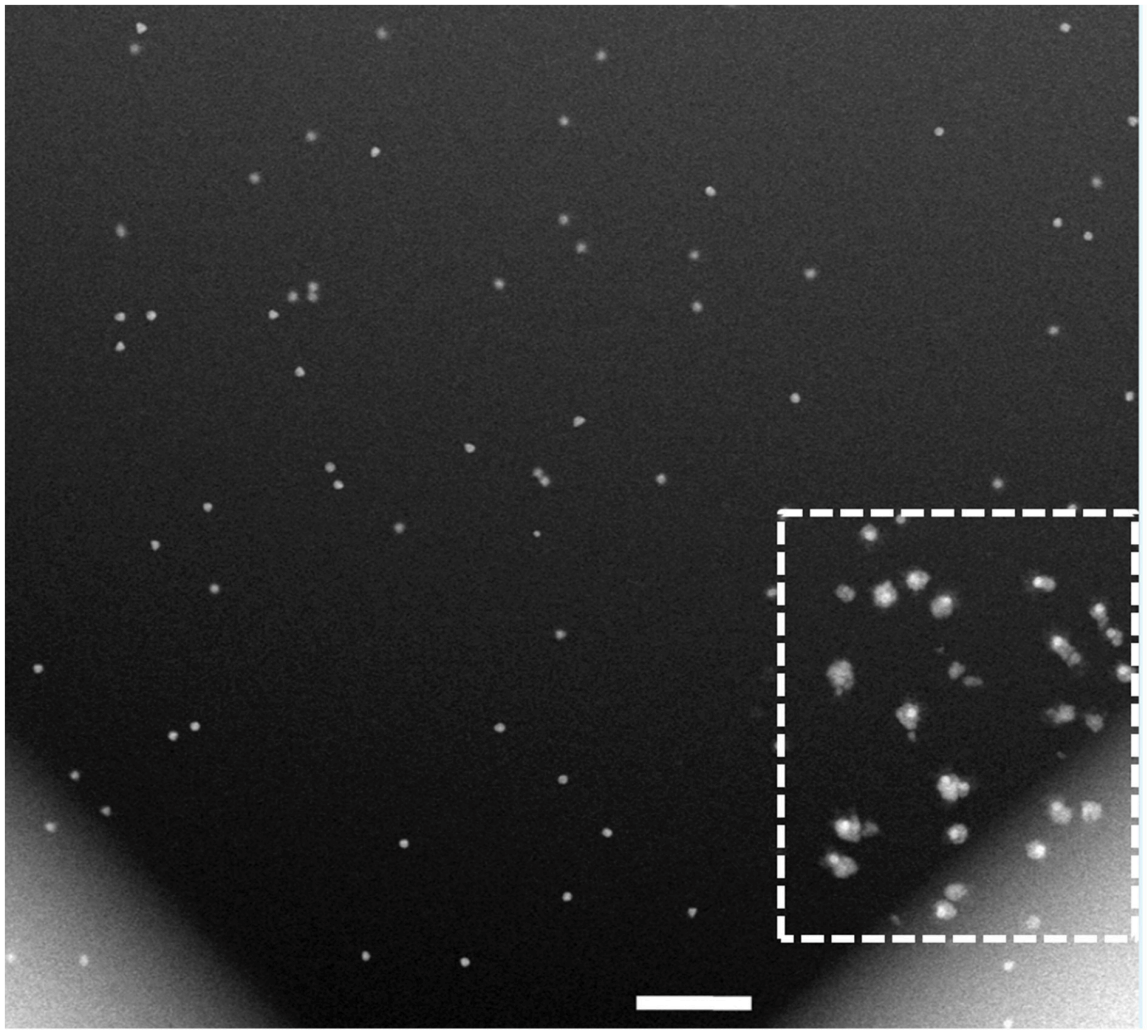
Low-magnification HAADF-STEM imaging of the cell after completion of the *in situ* analysis in Figures 3, 4. While the resolution in this low-magnification image is insufficient to detect and measure the Pd shell thickness, the region previously exposed to the e-beam, denoted with the dotted square, clearly shows the core-shell structures formed during the *in situ* reaction. The electron dose in the freshly exposed area of the fluid cell is calculated at 0.2 e^−^/Å^2^, and that of the previously viewed area is at 70.2 e^−^/Å^2^. Scale bar: 500 nm.

To elucidate the effect of electron beam onto the growth of CS NPs, we imaged a larger area at a lower magnification (20,000 *X*), as presented in Figure 6. This permitted comparison of two distinct regions: one that was previously exposed to the electron beam and another that was not imaged before, along with the respective electron doses received by the different parts of the viewed area. The low magnification STEM image obtained after acquiring several STEM images at 56,000 *X* magnification (boxed area in Figure 6), revealed that the CS structures were localized to the areas previously exposed to electron beam. It is likely, that the resolution in this low-magnification image is insufficient for detecting formation of the Pd shell in the rest of the viewing area, as the uniform growth of Pd shell around Au core was expected in all areas in the reaction chamber irrespective of electron beam presence. It is worth noting, that the field of the *in situ* fluid cell STEM does not have a standard cumulative electron dose on the sample similar to that established by the cryo-EM community. The dose rates in our experiments, however, place our work in the low dose regime (Supplementary Tables 1, 2; Woehl et al., 2012).

**Scheme 1:**
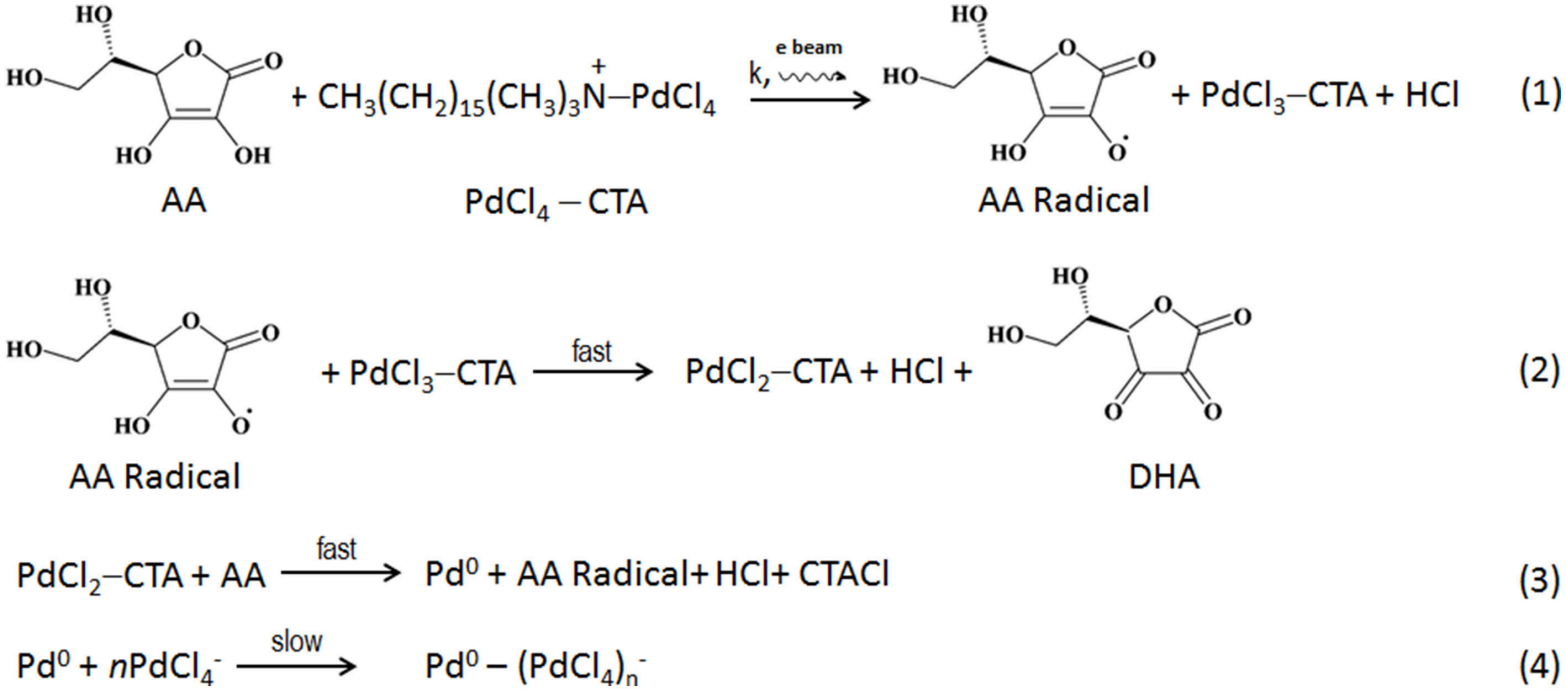
The suggested reaction pathway taking place in a liquid phase *in situ*.

Based on the acquired data, we suggest the following mechanism of Pd reaction with ascorbic acid in the fluid cell *in situ* (Scheme 1). The initial rate-limiting formation of ascorbic acid radical (Equation 1) is followed by fast 2-electron reduction of Pd precursor (Equations 2, 3; Khan et al., 2013; Li et al., 2013; Zhang et al., 2014). While we could not monitor concentration of the reagents during the *in situ* imaging, we hypothesize that the beam-induced radiolysis facilitates the formation of ascorbic acid radical, thus leading to the overall enhancement of the Pd reduction. The reduced Pd is deposited onto the surface of charged Au nanoparticle seeds, resulting in a formation of Au-Pd core-shell structures. Considering the decrease in the thickness of Pd shell along the <111> direction and its increase along the <100>, the rearrangement (i.e., coarsening and ordering) of the formed juvenile Pd shell likely becomes the new rate-limiting reaction step in the *in situ* reaction (Equation 4). Such a rearrangement is affected by the lattice mismatch at the interface region and the associated strain release, along with the surface reconstruction (Equation 4).

Importantly, despite maintaining the low dose STEM conditions, the electron beam interacts with the liquid contained in the reaction chamber, leading to formation of highly reactive radiolyzed species (Woehl et al., 2012; Abellan et al., 2014; Schneider et al., 2014; Park et al., 2015c). Along with regular radiolysis products, such as ${\text{e}}_{\text{h}}^{-}$, H^·^, OH^·^, H_2_, H_2_O_2_, H_3_O^+^, HO_2_^·^, electron beam radiolysis produces ascorbic acid radicals (AA^.^). This increases the rate of (Equation 1), thus affecting the overall reduction kinetics of the system. In the absence of electron beam, the reaction proceeds more slowly: this can be seen in the region not previously exposed to the electron beam. Here the formation of CS takes longer time and is not directly evident from the low magnification image. Moreover, the complex reaction products induced by the interaction of radiolyzed species with AA, are likely to affect the rate of formation of CS NPs. In addition, the cumulative electron dose delivered to the scanned area introduces additional electrons, and that might also contribute for such features present. The Pd shell growth corresponding to cumulative electron dose is presented above in Figures 4B,C reveals that the Pd shell growth takes place only in the presence of AA. The radiolysis of aqueous AA solution by the electron beam produces AA radicals along with other radiolysis products, and the core-shell formation occurs rapidly even under the low dose conditions.

**Figure 7:**
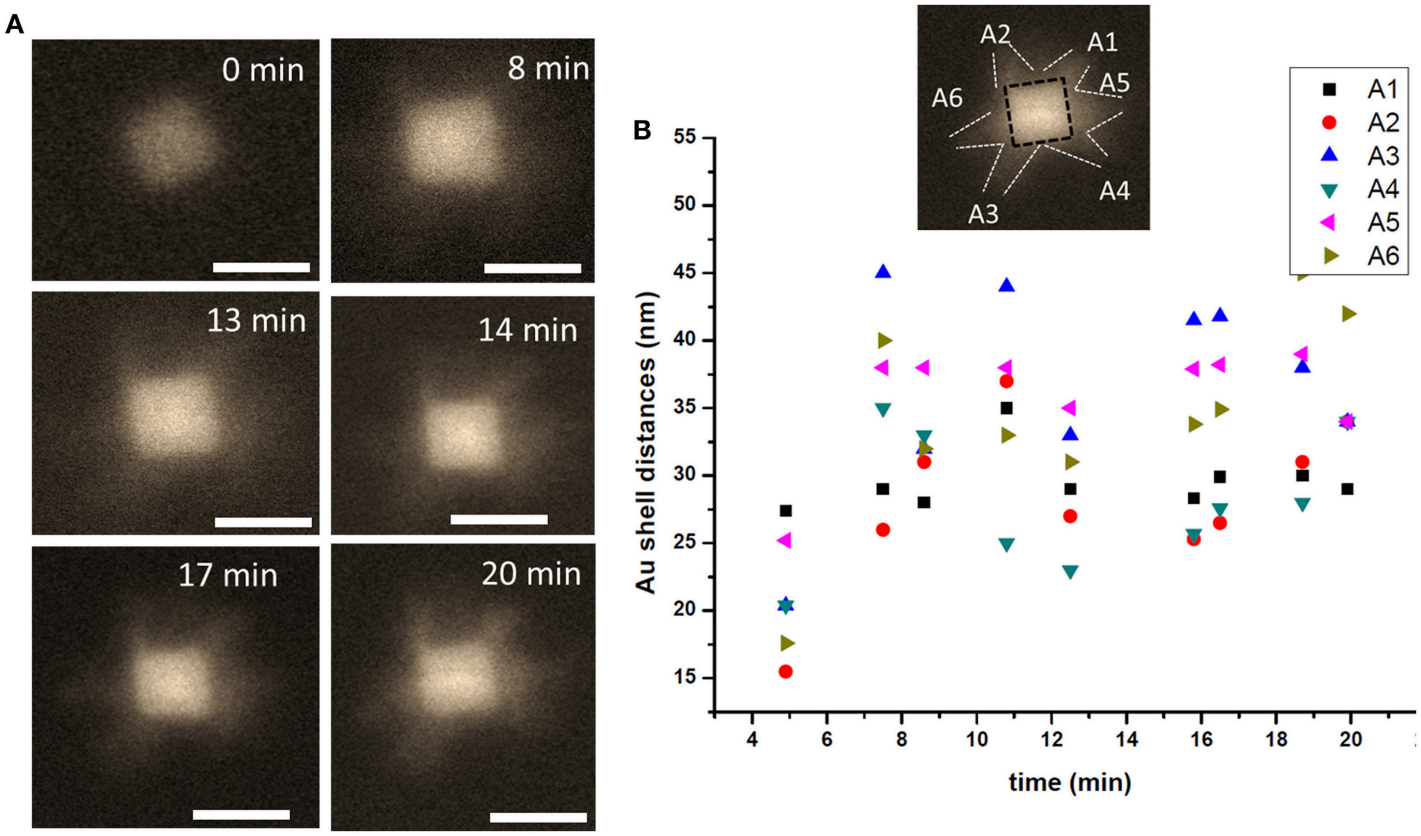
A nanostar in making. **(A)** HAADF -STEM image of the particle during the early stages of the Au shell directional growth process. **(B)** The particle growth is plotted as a function of time shown along the directions marked 1 through 6, with the shell thickness measured along the projected facet in every frame used for the image analysis. Scale bar: 50 nm.

We further utilized liquid phase STEM imaging to study DNA-directed shape transformation of core-shell nanocubes. An example of shape transformation using DNA oligomer is presented in Figure 7. Au-Pd core-shell nanocube is mixed with T30 DNAs, additional Au ions are introduced into the reaction chamber and the growth process is directly visualized in liquid phase. As shown in Figure 7, the growth proceeds from the vertices of the nanocubes and results in a nanostar- like morphology. The newly supplied Au ions reside onto the vertices with higher energy sites {111}, likely due to the weaker binding affinity of T30 DNA oligomer (Satyavolu et al., 2016, 2018) with nanocubes. This results in a directional growth, as can be seen in the snapshot acquired at 8 min of imaging. Starting from the vertices of the nanocubes, the deposition of metal ions leads to enhanced growth in these regions, as shown in the snapshots acquired at 14 and 17 min, and the nanostar-like morphology is observed ~20 min into the imaging. It is worth emphasizing, that since the imaging is carried out under the low dose conditions (i.e., low magnification) under the active fluid flow, the image resolution is somewhat impacted. For example, the contrast of the outer Au, which is influenced by its thickness, does not appear strong. Additionally, the concentration and pumping speed of AA were likely not fully optimized to have resulted in producing the optimal imaging contrast and/or final nanoparticle morphology. The rate of growth along different directions marked by A1 to A6 is measured and presented in Figure 7B and shows that the growth rate is different along different directions but the shell thickness increases as time passes by with the deposition of additional amounts of Au. For more details, we refer the Reader to Video 2. The “encoding action” by DNA oligomers here is manifested in directing the deposition and growth of outermost shell of metal. The result is in agreement with the recent report by Lu and co-authors (Satyavolu et al., 2018).

## Conclusions

Using the continuous flow fluid cell STEM holder platform, we have visualized formation of Au-Pd core-shell nanostructures in the presence of ascorbic acid in liquid phase *in situ*, while maintaining low-dose imaging conditions. The *in situ* synthesis was carried out in the near-native reaction environment resembling those in the solution synthesis, however the interaction of electron beam with AA in the liquid cell augmented the formation of AA radicals, which led to the enhancement of Pd precursor reduction and facilitated the deposition of the Pd shell over the Au core (Dispenza et al., 2015). Compared to the solution synthesis reaction, the proposed mechanism of the formation of Au-Pd core-shell structures *in situ* involves rapid formation of AA radical and initial reduction of Pd ions (Equations 1–3, **Regime I**), followed by a slower rearrangement of Pd shell to form an ordered structure (Equation 4, **Regime II**). The newly deposited Pd ions undergo rearrangement, yielding a fully formed core-shell structure. Hence, the growth mechanism of formation of core shell can be explained by the complex phenomena that do not obey any one classical theory. From the experimental point of view, controlling the delivery of liquid reactants (Pd precursor and AA) in a reaction chamber in these experiments remains a real challenge with a spatial distribution of reagents in the region of interest likely non-uniform, thereby affecting the overall processes.

While we strived to recreate the native synthetic conditions in the reaction chamber *in situ*, we could address neither the complex phenomena associated with the dynamics of the liquid flow in constricted volume of the reaction chamber with bowing window membranes, nor the highly debated diffusion of reagents in a nanofluidic regime (Abellan et al., 2014; Schneider et al., 2014). Additional factors, including the beam-induced production of hydrated electrons, radiolysis of AA, and direct reduction of Pd ions, will need to be examined further when considering reaction pathways. However, while the growth of the Pd shell is influenced by these factors, we successfully captured the early stages of the shell formation of the core-shell Au-Pd nanostructures and established the mechanism of their growth *in situ*. We concluded that hydrated electrons and enhanced formation of AA radicals affect the entire CS growth mechanism by altering the reaction kinetics. The enhanced reduction of Pd ions, which promotes the initial deposition and rapid anisotropic growth of Pd, yields to a slower orientation and redistribution of disordered Pd to produce an ordered and compact shell. This occurs *via* a process similar to oriented attachment and becomes the rate-determining step in the *in situ* reaction. In addition, the particles are free from translational motion but presents some rotational or wobbling of individual particles. This rotational or wobbling motion of nanoparticle needs to be explored more in order to understand the particle growth along certain crystallographic axes determining the growth of shape controlled NPs. Finally, we showed a direct shape transformation for DNA-templated NPs exhibiting the shape transformation from Au-Pd core-shell nanocube into Au-Pd-Au core-shell nanostar-like morphology. Our findings reaffirm that S/TEM imaging in liquid phase *in situ* is applicable to creating near-native reaction environment within a confined space of a fluid cell. When the imaging is performed under the low-dose conditions, this method can be used for a direct visualization of early stages of formation of complex nanostructures, not observable otherwise.

## Data Availability

The datasets generated and analyzed for this study can be found in https://doi.org/10.25380/iastate.c.4175264.v1

## Author Contributions

NB designed and carried out the experiments. Both authors participated in discussing results, interpreting the data and writing of the paper.

### Conflict of Interest Statement

The authors declare that the research was conducted in the absence of any commercial or financial relationships that could be construed as a potential conflict of interest.

## Supplementary Material

The Supplementary Material for this article can be found online at: https://doi.org/10.25380/iastate.6850991.v1 and https://doi.org/10.25380/iastate.6851063.v1
